# A Struggle for Survival: Meaning of Late Life in a Rural District in Uganda: A Qualitative Study

**DOI:** 10.3389/fpsyg.2021.699485

**Published:** 2021-08-05

**Authors:** Birthe Loa Knizek, Davy Vancampfort, Japheth Kwiringira, Elizabeth Kyazike, James Mugisha

**Affiliations:** ^1^Department of Mental Health, Norwegian University of Science and Technology, Trondheim, Norway; ^2^KU Leuven Department of Rehabilitation Sciences, Research Group for Adapted Physical Activity and Psychomotor Rehabilitation, Leuven, Belgium; ^3^Department of Sociology and Social Administration, Kyambogo University, Kampala, Uganda; ^4^Department of History and Political Science, Kyambogo University, Kampala, Uganda

**Keywords:** meaning, Africa, old people, elderly, poverty

## Abstract

**Background:** Between 2015 and 2050, the aging population of Uganda (aged 50 years and older) will be nearly doubled. Therefore, later-life problems have become an area of increasing research and policy interest. This study aimed at exploring how aging people living in extreme poverty in a low-income country experience their everyday life and what kind of meaning systems employed by them to understand and cope with their living conditions.

**Methods:** We conducted a qualitative interview with 14 participants in the Buikwe district. In this interview, 11 women and 3 men were included, and a thematic analysis was employed for data processing and analysis.

**Results:** Unanimously, all participants reported their condition as extreme poverty. The key informants (KIs) emphasized respect from descendants and the community as a foundation for a meaningful later life. In contrast, this aspect has been ever mentioned by no caregivers but by only one care-receiver. The willingness/ability of children to support the elderly who are in need of support formed a major part of the reflections of care-receivers, which would be decisive for their position in the society and the respect they would receive. In addition, both Christianity and traditional beliefs as well as beliefs in witchcraft and ancestral spirits were employed as a basis for actions and reflections.

**Discussion:** The question arises whether life in extreme poverty conditions can be perceived as meaningful. Respect was mentioned as fundamental by the KIs, thereby giving priority to social relations as the most meaningful factor for living a meaningful life. The ability and willingness of the possible descendants for support as the focus of care-receivers might be a more down-to-earth description of this aspect but without using the same level of abstraction. For the majority, due to their belief system did not serve as a source of consolation their main focus was on social relationships for support. To improve the wellbeing of the old people, their sense of meaning must be restored through a system, guaranteeing the coverage of basic needs and measures to restore dignity through a reintegration in both community and congregations. Social service agencies who are targeting the elderly people need to work toward this objective.

## Introduction

Getting old for many people includes significant social, personal, and/or physiological changes including the loss of physical abilities, relationships, and social position. Health becomes a central issue when physiological changes become noticeable and the risk for chronic diseases gets increased, which again could impair the quality of life and wellbeing. According to the report of WHO on aging and health, health must be considered as a “fundamental and holistic attribute that enables older people to achieve the things that are important to them” (World Health Organization, 2015, p. 27). Important ingredients for healthy aging are integrity, dignity, freedom, and autonomy, which all are collectively called as human rights (*ibid*). In addition, the United Nations (2006) Convention on the rights of persons with disabilities especially focused on the rights for all people with a particular form of functional limitation as most older people have acquired, to live, and to be included in their community. In these perspectives, the concept of “functional ability” becomes important. According to the WHO's International classification of functioning (WHO, 2001), functional ability must be understood as health-related attributes, which make it possible for people to be and to do what they have a reason to value. Functional ability is made up of both the intrinsic capacity of the individual and relevant environmental characteristics as well as the interaction between those. The net physical and mental capacities of an individual make up the intrinsic capacity, whereas the environment consists of the contextual factors influencing an individual. Structural, physical, political, and normative factors as well as health and social policies, healthcare services, and other systems as a potentially providing support are part of the context, which is central for the functional ability of an individual (WHO, 2001). This means that, in addition to the possibility of getting help due to increasing health issues, WHO emphasizes the support of intrinsic capacity/individual resilience based on the ability of an individual for a positive adaptation in case of adversities and the upholding of wellbeing and meaning in life. However, one has to differentiate between the ultimate meaning of our lives as a lifelong quest and meanings in life, which are everyday events or activities that individuals ascribe importance to, in accordance with their value systems guiding their behavior (Frankl, 1969/2014). Both meaning in life and meaning of life are of crucial relevance for wellbeing and health as “…the acute suffering of someone who lacks meaning in life can lead to stress, ulcers, and even suicide” (Baumeister, 1991, p. 30).

The meaning-making concept has attracted renewed attention and further development in the last 20 years (Park, 2005, 2010; Seligman, 2011; Proulx and Inzlicht, 2012; De Marinis, 2018). Meaning-making is essential for a successful adaptation to shifting environments and consequently must be understood as an important part of the intrinsic capacity, which makes up a central component of the functional ability. In the meaning-making model of Park and Folkman (1997), one distinguishes between global meaning that encompasses, for example, beliefs about life, life goals, or idealistic commitments, and situational meaning, which are meanings “…in the context of a particular environmental encounter” (Park, 2010, p. 258). The global meaning system is a construction of both accumulated personal experiences and cultural influences that include dominating belief systems (Baumeister, 1991). In this global meaning system, we find assumptions about control, predictability, goals, etc. (Janoff-Bulman and Frantz, 1997; Mischel and Morf, 2003; Park, 2005, 2010). The global meaning system can be challenged by, for example, injustices, loss of persons and abilities, illness, poverty, or catastrophic events, and the person must figure out whether the situation is threatening and/or controllable and which consequences it might have in the future (Park, 2010). If the person experiences a discrepancy between the global meaning system and an actual situation, he/she has two options: revise the global meaning system (accommodation) or the appraisal of the situation (assimilation). Both cognitive and emotional forces are employed in these processes (Hunt et al., 2007; Sloan et al., 2007), which consequently have been termed as cognitive-emotional processing by Hayes et al. (2007). The result of these processes can be described as having “made sense” (Davis et al., 1998) or “meanings made” (Park, 2010) and can include growth, predictability, or coming to terms with an event or a condition (Evers et al., 2001). Other perspectives on meanings made are reattributions and causal understanding (e.g., Janoff-Bulman and Frantz, 1997; Westphal and Bonanno, 2007), changes in identity (Gillies and Neimeyer, 2006), global beliefs (Park, 2005, 2010), or global goals (Martin and Tesser, 2006) or a reappraisal of the meaning of a stressor (e.g., Resick et al., 2008), where the implications of an actual situation are re-evaluated in a more positive light (Park, 2010). Meaning-making as part of the intrinsic capacity in the functional ability of a person consequently seems to be important for health and wellbeing throughout all stages of life, including the later part. Dominant religious belief systems seem to be helpful for many people to be comprehensive meaning systems (Hood et al., 2005; Silberman, 2005; Newton and McIntosh, 2013) offering meaning and hope in a variety of situations (Hall and Hill, 2019). This is especially relevant in Uganda, a highly religious country in which the majority of the people (about 84%) are Christian, 14% are Muslim, and the remaining people belong to traditional African religion (Uganda Bureau of Statistics National Population Housing Census, 2014).

The existing literature seems exclusively focused on older people in resourceful settings, whereas settings with limited resources have been overlooked despite the fact that most people in the world live in poverty (Worldometer, 2021). In addition, the effect of the worldwide AIDS epidemic, which mostly occurs in the developing world, has received relatively little consideration with respect to older persons despite their lives being significantly affected in various ways (Knodel et al., 2003). The purpose of this study was to explore how HIV-infected older persons in a low-income country experience their everyday life and what kind of meaning systems are employed by them to understand and cope with their living conditions. As meaning-making seems related to health and wellbeing, this kind of knowledge might be important for the improvement of the conditions for the elderly in Uganda.

### The Setting of the Study

Uganda is a low-income country with a young population. Almost half of the population of Uganda estimated to be 46,581 million is less than 15 years old and only about 10% are <45 years, and the median age is 16.7 (Worldometer, 2021). While Uganda has adopted the UN definition of older persons as those aged 60 or more years, WHO operates with a figure of 50 or more years, resulting in some uncertainties about the data officially collected due to a variation in the data. From 2016 to 2018, the percentage of people aged 60 or more in Uganda increased from 2.9 to 3.7% (Uganda Bureau of Statistics, 2018). In 2020, the life expectancy at birth for men was 62 years while the same for women was 66.7 years (Worldometer, 2021). Most of the older people are living in the countryside with limited economic resources and access to healthcare (Ajiambo, 2016). About 85% of the people are extremely vulnerable as they are doing crop farming and have no formal social security. Uganda also has a high burden of people living with HIV, which is reported to be 1.46 million in 2019 (Uganda AIDS Commission, 2020). Elderly people might be both infected and affected as HIV/AIDS has claimed its victims and left orphaned children behind in the care of the elderly (Ministry of Gender Labour Social Development, 2018). Rapid urbanization has contributed further, and many parents have left for the city while the children traditionally are sent to the grandparents who are in the countryside for helping their grandparents (Ministry of Gender Labour Social Development, 2018). However, this implies further economic pressure as the parents seldomly contribute and the grandparents are left with the responsibility of providing the children with food, clothing, school fees, etc. (Ministry of Gender Labour Social Development, 2018). Without means, the elderly are captured in a situation of chronic poverty, which counteracts wellbeing:

Poverty and well-being are two sides of the same coin. Old age brings with it a higher risk of poverty in countries like Uganda, where the majority are poor throughout their lifetime. An average older person has survived traumatic periods of political upheavals, war, natural disasters and loss of wealth. In old age, they find themselves still living on low incomes while lacking proper attention when they are ill or develop disabilities (Ministry of Gender Labour Social Development, 2018, p. 18).

Under the Social Assistance Grants for Empowerment Scheme (SAGE), the Ugandan government started, in 2015, to implement the Senior Citizens Grant and to roll it out successively to an increasing number of districts. The pension targeting senior citizens aged 65 years and older amounts to 25,000 UGX per month, which equalizes about 7 US$. This pension program was rolled out in 55 of 135 districts of Uganda (Uganda AIDS Commission, 2020) but is intended to cover the entire country. Momentarily, most people do not receive this pension and in addition, it is difficult to retrieve the pension and it does not always reach those, who might be entitled to it and the funds can delay for several months. Empirical evidence reveals that only 7.1% of the older people have access to pension, of which 60% are men (Ajiambo, 2016). As a result, the elderly must rely entirely on informal networks:

In Uganda, a person's safety net is their extended family as well as their community. These relations make up the “kinship networks” which offer them financial, physical, and emotional support, and a person becomes vulnerable when these are weak. Care is reciprocal so one must often contribute and look after others in the hope of receiving care in old age (Ministry of Gender Labour Social Development, 2018, p. 6).

In addition, one has to consider social norms, which give men the right to remarry, while the women do not. Many men leave their older wives and marry younger women, who can take care of them in old age, and it is not uncommon for a man to have more than one wife (Najjumba-Mulindwa, 2003). Contrary, older women are dependent on other's willingness to support and care for them, simultaneously, twice as many older and must continue to work as long as possible despite any physical impediments to avoid dependency and unpredictability (Ministry of Gender Labour Social Development, 2018). Older women consequently are left in a very vulnerable situation with accumulated disadvantages (Najjumba-Mulindwa, 2003; Rishworth et al., 2020) and receives less support than men in every area. The Senior Citizen Grant would be helpful but does not reach the majority of women and might not even be intended for easing their situation as the (Ministry of Gender Labour Social Development, 2018, p. 2) in a pamphlet on aging states the following: “An old age pension, for example, allows more children to attend school. It enables a person to age gracefully while delaying dependency.” Obviously, the limited pension is not only meant for the benefit of the old person but also as a means to support children, so that the old people can fulfil their social duty and uphold dignity and respect.

Furthermore, religion is an important factor to consider in the central region, where Buikwe is located, as 41.2% inhabitants are Catholic, 30.1% are Anglican/Protestant, 5.9% are Pentecoastal, 1.9% are 17th Day Adventists, 0.2% are Eastern Orthodox Christian, 0.8% are Other Christians, 18.4% are Muslim, and 0.1% are Traditional (Uganda Bureau of Statistics National Population Housing Census, 2014).

## Methods

### Participants and Procedure

This was an exploratory qualitative study on meaning-making among the elderly in Uganda. This study was part of a pilot study on: the efficacy of physical activity counseling in Ugandan patients with HIV and a comorbid mental disorder in the Buikwe district. The project was being implemented by African Social Development and Health Initiatives, a local NGO in Uganda. This substudy aimed on old age and depression and aimed at exploring how elderly living in extreme poverty in a low-income country experienced their everyday life and what kind of meaning systems employed by them to understand and cope with their living conditions later in life. The overall theme was how indigenous communities age with depression, and this was discussed along with other variables such as general poverty, access to basic social services, and health, gender dynamics, factors that facilitate or hinder access to basic social services in the context of having mental disorders such as depression. Through this funneling method, a lot of information was generated, and other themes and subthemes could emerge.

We conducted semi-structured interviews with 14 participants in the Buikwe district. The participants were elders who lived in the area for a long time practicing both Christian (Catholic and Protestant) and traditional religion and attended congregations on a regular basis. To cover the material and normative aspects in addition to the living experiences, we purposively recruited participants in different positions who had an intimate knowledge of aging in the district. Five of the participants were key informants (KIs), who had different formal and informal functions as elders/community leaders and were in the age range of elderly related activities. They were actively involved in community development activities by virtue of being elders. Quite a number were also community mobilisers and played leadership roles in their congregations. Four participants were care-receivers and five were caregivers. Due to the low life expectancy and some insecurities on the definition of elderly (WHO 50+ years, UN 60+ years), the elderly care-receivers were aged between 52 and 62 years. The caregivers were aged between 32 and 52 years while the KIs were aged between 51 and 65 years. Of all 14 participants, only 3 female caregivers were less than the age of 50 implicating that the remaining participants all belonged to the group of elderly people. We interviewed 11 women and 3 men. Among the men were one caregiver, one care-receiver, and one KI.

### Data Collection

We approached the district leadership in the Buikwe district on the selection of the study subcounty. We chose a subcounty (Buikwe subcounty) where there are activities targeting elderly people in the district from both the public and private sector organizations. We approached the subcounty leadership to help us in the selection of community leaders who were engaged in the activities meant for the elderly. The community leaders had in turn helped us to select the elderly people who are in their communities. Data from the elderly people were collected by trained research assistants while all the interviews of KIs were conducted by the last author, who is a trained social worker and has worked with poor and remote communities for over 25 years. He is well-trained, has skills and competencies in community immersion, and potentially speaks at the level of the participants.

All interviews were conducted in the local language (Luganda). Interviews for elderly people were conducted at their homes while all the KI interviews were conducted in an office at Buikwe Health Centre III. Both KI interviews and elderly interviews lasted between 45 min to 1.5 h. All interviews are taped, recorded, and transcribed, and translated into English by a research assistant, who is from the same culture. The interviewer is a mental health worker and senior academic based on Uganda, speaking both English, and Luganda, the local language spoken in the area. The translator is a university graduate, who is born in the same culture, is grown up in a rural setting, and speaks fluent English and Luganda.

### Analysis

The interviews of KIs were in general both longer and richer than the other interviews and covered more topics as relevant for aging. The participants had varying experiences in dealing with outsiders such as researchers. The KIs were more experienced (by virtue of being in community work for a long time) and spoke more than the others. The interviews with the care-receivers and caregivers would probably have been longer if we had time to spend in the field with them. Due to the perceived large differences between the KI interviews and the other participants, we decided to analyze the KI interviews separately and use the information gained from them as a background against which the interviews from both the caregivers and care-receivers could be analyzed. This resulted in the interviews of the KIs being analyzed solely by means of thematic analysis (Braun and Clarke, 2006) while the remaining interviews were analyzed through a directed thematic analysis (Hsieh and Shannon, 2005), where the analytic process is not only guided by the themes constructed through the interviews of KIs but also allows new themes to emerge. We chose this procedure as the remaining interviews were quite sparing of words and had to be interpreted in the context that the KIs had revealed based on their overall knowledge of existing conditions and normative context. The method of Braun and Clarke (2006) employed by us for the analysis of the KI interviews has proven as a flexible method, which can be employed within a variety of paradigms. Six phases of thematic analysis aim at capturing patterns and meanings expressed by the participants. The initial phase of familiarization with the material is succeeded by phase two and three, where the interesting features are detected and coded and themes developed based on the codes that seem to belong together. In the consecutive phases, the initial themes are checked and reviewed against the material, and final themes are developed in a recursive process (Braun and Clarke, 2006) moving back and forth between the phases as well as the data and our increasing understanding of them until final themes are reached. These themes were then used further in the next step where we analyzed the remaining interviews with a directed thematic analysis (Hsieh and Shannon, 2005). The process is quite like the process of Braun and Clarkes except that one reuses the themes from the analysis of the interviews of KIs. At the end of the process in both the approaches, the themes are quality checked against the text to control whether everything is captured.

### Ethical Considerations

Ethical approval for the project was secured from Mengo Hospital Research Ethics Committee as well as from the district, subcounty, and Buikwe Health Centre III management. All research assistants were trained in ethical issues (such as climate setting, informed consent, and confidentiality) related to the study before data collection. All participants were informed of the objectives of the study and provided oral consent before the interviews. They were assured of confidentiality and that all data collected were kept under key and lock, only accessible to the research team. To protect the anonymity of the participants, they will be referred to as KIs with number and gender (for example, KI1F), care-receivers (for example, CR1F), or caregivers (for example, CG4M).

## Findings

### Perspectives of KIs

The KIs were four women and one man aged between 51 and 65 years, which makes them members of the group of elderly. The KIs all had a position as counsellors or a position at the village health team where they both had knowledge about the conditions of the elderly and social reactions as a result of the normative context. Their knowledge not only included rights but also shortcomings from both government and community. Their statements included the conditions of getting old today compared to the former times regarding support and the social reactions toward the elderly. One prominent, thoroughgoing topic in these interviews was the notion of respect, which was perceived as the fundamental of experiencing a meaningful life. The other prominent theme was supernatural powers and the relationship with them.

### Respect

Respect for old people was underlined as an important factor for living a meaningful life in the interviews of all KIs, which is in concordance with the claim of Mbele et al. that respect is an essential component of African culture forming the foundation for existence:

…African people have always recognized respect, as a concept, experience, and practice with spiritual and cultural dimensions of great breadth, depth, and height. Such practice is recognized as crucial for the promotion of local, international and global health, and well-being (Mbele et al., 2015, p. 87).

The fundamental role of respect must be seen in relationship to the African, holistic worldview, which is still highly influential. Everything is part of the same universe and interdependent, which means that a person is nothing without being part of a social order/community. Consequently, “…an individual is obligated to contribute to the community not because it is expected of him or her, but because it is him or her (…) If one does not devote oneself to the welfare of the community, in a sense, one purposely causes harm to oneself” (Verhoef and Michel, 1997, p. 396).

In line with this, the KIs emphasized that respect was not something, which one could expect automatically when getting older. In a society where respect is the foundation of existence and an essential component of meaning-making, lack of respect has huge consequences. Some of the KIs noticed a difference from former times and they claimed that it was better to grow old in the past: “Being old of long ago was nice more than the one of now days” (KI2F). This respondent bases her opinion on her perceptions of children caring less for old people in addition to community members more sticking to their own families and not taking responsibility for others. This must be regarded as a severe breach of traditional communitarian norms, where respect, care, and responsibility for other people especially from own community are fundamental and installed from early childhood in the mind of the community members (Mafunisa, 2008). Moreover, the traditional norms are described as doctrinaire and dogmatic as “…the communal context and authoritarian structures, in terms of the respect that is accorded to elders and traditions, also indicate that these beliefs cannot be questioned and critically examined” (Ikuenobe, 2006, p. 257). The disruption of the family support that our respondents note is quite dramatic and has been described both as a result of the HIV/AIDS epidemic (Knodel et al., 2003; Seeley et al., 2009) and rapid urbanization leaving old people alone at the countryside (Apt, 2001; Aboderin, 2006).

Our respondents agree that aging was easier previously and give as reasons the HIV/AIDS epidemy: “The elderly of long ago was very well off, all these sicknesses had not come in those people” (KI3F). According to this lady, the problems that the elderly face is related to sicknesses, which did not exist earlier. From the context, we can deduce that she is referring to HIV/AIDS and the complications related to being infected, which have a considerable impact on the possibilities of being respected and live a meaningful life. HIV/AIDS both can be stigmatizing due to presumed sexual activities of the patient and the inhibitory physical consequences. In addition, old people can be affected by HIV/AIDS by becoming the caretaker of orphaned grandchildren and relatives, which contributes to the economic burden and worries about them: “If you worry a lot, you grow old very fast” (KI1F).

### Who Will Be Respected?

The question of who will be respected is closely related to what is regarded as respect. KI1F mentions that elderly people are normally referred to in a respectful way (Jjaja), but all KIs agreed that respect is an interpersonal phenomenon, which presupposes some efforts from the old person him/herself:

I: Hmmm, do you think growing old gives respect?
R: There are people who grow old with respect, but you can even work for your respect.
I: So, you must work with your respect, you don't grow old with it; me, I thought if you are old, every elderly is respected? That growing old is respectable.
R: You work for your respect. (KI4F)

The perception of how one can work for respect seems to differ somewhat between the KIs. KI3F is very clear about the conditions of getting respect: the child has bought him/her a car and she/he has money and property. If somebody on the contrary has no means for their own support as food, she/he will lose every respect. In sum, this means that you have to be wealthy, have children that are willing and own the means to support you as a prerequisite for respect and the possibility of living a meaningful life. The arguments of the KIs seem to be somewhat circular as an old person gets respect when looked after but is only looked after in case of having respect. KI5M seems to unlock this circle by explaining that one gets respect when one has prepared for old age and loses respect if one has failed to prepare for old age. The preparation implies ensuring financial independency and the upbringing of descendants, who can ensure necessary care: “An elderly to be with respect is when he educated his children” (KI2F). Education of the children means here both an investment in the basis for a meaningful old age as the education both gives the descendant job possibilities and access to financial means, and the installment of an accept to accept the obligation to help parents when necessary in old age.

To clarify further the question concerning respect, KI1F gives an example of an old lady who has no respect at all in her community because of her situation and behavior. Her fundamental problem is that she is childless and never gave birth. This is already problematic because this is against the norms. It also implies that she has no descendants, who would be able to care and provide for her. In addition, one way of earning respect is to bring up children who behave well and according to the normative expectations. She missed that chance too. According to KI4F, the lady publicly proclaims that she is not ashamed of not giving birth and abuses other community members. As she also drinks alcohol and hangs around with young boys, she has violated so many normative expectations so that she is not respected by anybody, which probably worsens her situation even more. Also, the other KIs talk about disrespect to old people caused by misbehaving, which, according to them, means drinking alcohol, talking nonsense, or being abusive.

Another issue influencing respect is the health status of the elderly. If you need to walk with a stick, the respect seems to disappear according to KI1F. In addition, deteriorated health is perceived more common today than earlier: “The ones of long ago didn't get sick all the time, or what? But now they get sick a lot” (KI4F). Unhealthy food, increased use of chemicals in farming, and the loss of property are the perceived causes of more pressure and sickness, which then again implies the loss of respect from the other community members. AIDS is never mentioned in relationship to respect, which might be seen in relationship to Uganda's work in the reduction of stigma as official figures show a reduction of external stigma and discrimination from 24% in 2013 to 1.3% in 2019 (Uganda AIDS Commission, 2020).

However, health issues traditionally mean more than an individual problem:

Health does not simply mean the absence of disease; it incorporates balance and harmony between the individual and his or her social surroundings, including harmony with self. Disease results from the breakdown in relatedness, including disharmony between the individual and the rest of the universe (Mkhize, 2008, p. 39).

Traditionally, health issues are regarded as disharmonious interpersonal and intrapersonal relationships and consequently carry a negative meaning. KI4F touches on the necessity of intrapersonal harmony in her statement: “You first give yourself respect and in your old age people say Yiii, that woman has grown old, but she has grown old with her respect.” She elaborates further on how one can give oneself respect by helping others. She seems to be in line with the observations of Mkhiz by underlining the mutual dependence of internal and external harmony. However, the normative system also installs limitations to who might be able to help. KI2F tells, for example, about a lady who was incontinent and defecating on herself. The person, who usually would help her, was a nephew and based on the normative context regarded as in-law and therefore forbidden to help her with personal hygiene. This is an example of how the normative system even might limit the possibilities for the reception of fundamental, personal care even further. Apparently, only the self-reliant elderly can be respected and dependency on others can be interpreted by community members as a personal flaw and character weakness implying that one must prepare adequately for his/her old age. This is interesting as Uganda is regarded a highly religious country (Uganda Bureau of Statistics National Population Housing Census, 2014). In the central region, where Buikwe is located, 41.2% are Catholic, 30.1% are Anglican/Protestant, 5.9% are Pentecoastal, 1.9% are 17th Day Adventists, 0.2% are Eastern Orthodox Christian, 0.8% are Other Christians, 18.4% are Muslim, and 0.1% are Traditional (Uganda Bureau of Statistics National Population Housing Census, 2014). Mercy and compassion are the main virtues in the religions that almost all Ugandan confessed to. These virtues also fit very well with the traditional communitarian norms that fostered care and support for fellow community members (Ikuenobe, 2006; Golaz et al., 2017). Based on the statements of the KIs, it does not seem that the contemporary elderly are necessarily included in these considerations. The shortness of resources seems to have affected which persons have a priority for support.

The Ugandan Government has recognized the financial hardship of old people and is in the process of rolling out the Senior Citizen Grant, whose “… aim is to provide all older people in Uganda with a regular pension based on their rights as citizens, and in recognition of their contributions to the nation over their lifetimes” (Ministry of Gender Labour Social Development, 2018, p. 20). Despite disagreements on the speed and the fairness in which these grants reach people, one also can find disagreements on the suitability of the usage of finances for this purpose. On questions whether public financial support would relief the situation of the elderly, for example, KI5M answers:

R: … it would be good, but that money doesn't help them.
Ii: Hmmmm
R: Because it would have been, now like a person of 19 years, 20 years can use it.
Ii: Hmmmm
R: And they work, and they develop.
Ii: Hmmmm
R: But this person just eats it.

In the opinion of this male KI, financial support to the elderly is a short-sighted investment. While young people would be able to develop a living and a family and be an asset for the community, food for the elderly is perceived as a waste of resources despite religious and communitarian norms of compassion. It is therefore no surprise when KI2F talks about the children only waiting for the old to die, so that they can inherit their possessions. According to these KIs, the traditional inclination to support the elderly has vanished in the context of chronic poverty and some question the suitability of the allocation of resources by the government for the support of the elderly people. The situation is complex because government pension scheme seems to be helping more the dependents of the elderly than the intended users. It is natural to assume that establishing meaning in later life might be difficult under these circumstances.

### Supernatural Powers

Dominating belief systems influence the personal worldview, which contributes to coping in the context of encountering difficult life events (Hall et al., 2018). Paloutzian (2005) states that the meaning-system cannot be understood independently of some faith elements. Being a very religious society, it is not surprising that supernatural powers as a basis for meaning in life are mentioned frequently by the KIs. Meaning in life is to surrender to God's will as “God plans everything, not you” (KI3F) and “It's God who decides” (KI4F). Besides a strong tendency to blame the elderly for their own misery, the KIs attribute everything to God and understand the meaning of life as being part of a divine plan. In addition, all the KIs talk about witchcraft as a vital factor for meaning-making among people in general and the elderly in particular. On the one hand, the elderly might be accused to be witches: “On the village they say that old lady, aaahhhhaaa, she has finished all the people in this village. She has bewitched all children in this village, aahhhaahhhaaa” (KI1F). In cases like this, the situation becomes even more serious for the elderly as people will avoid him/her totally because witches are known to have the power and will to kill. In addition to being blamed for their own misery, the potential risk of being blamed for the misery of others and misfortune and perceived as evil minded. Especially, women seem in danger, according to KI5M, as barrenness might be interpreted as a sign of being a witch. On the other hand, the elderly themselves grasp quickly the notion of witchcraft to find meaning in their situation according to KI4F. If their children who were supposed to look after them have died, very soon witchcraft thoughts are presented as meaningful explanations, and the remedies to counteract further actions from this evil force are sought for at the witchdoctor.

The statements of the KIs are important as all of them have an intimate knowledge of the conditions and beliefs of both the elderly and the community's opinions of the elderly. Through their positions and daily interactions with the elderly, they have a nuanced opinion about the conditions for experiencing meaning in late life, at the same time as they are in the same age group. Their statements provide the socio-ideological context for analyzing both the care-receivers and caregivers in their attempts to make meaning of their emaciated conditions.

### Perspectives of Care-Receivers

The care-receivers were all without a job except one lady, who sometimes could sell some leaves in the possibility of making a living. Of the four participants, one was a man and he was the only one not living alone. All care-receivers aged between 52 and 62 years and were HIV positive. The interviewers especially attempted to find out what the participants knew about depression and how to avoid it. In these interviews, the participants revealed what they perceived as meaningful in their life and how they employed their global meaning system to understand and approach problematic situations.

While respect as the foundation of a meaningful life was very prominent in the interviews of the KIs only the male respondent talked about this. Maybe this must be seen on the background of his gender and be the only one experiencing respect. He was 53 years, had one wife and six children. He used to help himself financially, and dependency is a problem for him as it influences his dignity: “…now I feel like a beggar because there is nothing, I can do for myself….” On the other hand, he carefully elaborates on “the required respect” that he receives:

C: Well my younger brother gives support and gives me the required respect and checks on me and also confides in me
I: How about neighbours?
C: They also respect me and greet me once in a while, so we work together

The respondent seems to be convinced that he is entitled to respect as he talks about the required respect. The respect he receives contributes to his wellbeing and he feels satisfied with the situation:

C: They do come and check on me, and we talk, and I feel happy
I: And how do you feel? Meaning that being around people helps!
C: I feel good and cared for and it reduces the thoughts and worry

In contrast, this man lives together with his wife and four grandchildren, who look after him, and seems integrated and respected in the community. The brother and children support him financially and pay him respect. His wife takes care of all his needs, including the management of his illness. He feels well, and life is meaningful as he still feels like a valuable and an integrated member of the community as people around him accord respect.

The women, on the contrary, all live alone and do not receive care or support regularly. The reasons for their living alone, which they often describe as loneliness, are different. CR1F is a widow of 54 years living alone because she only has daughters who are married now, and her grandchildren are at school. She has 2 years of schooling. She sells occasionally leaves on the market to be able to afford salt and food. On a question, whether she ever felt depressed, she answers: “No, like having thoughts about being sick and all that, I don't have them. The only thoughts I have are those that come from being alone and no one to take care of me.” She has been on HIV treatment for 10–20 years and is less worried about this than being alone. It is, however, not clear whether she feels lonely or short of help and financial means to avoid worries: “Well it helps if you have grown up children working in town and bring you all necessities and financial aid, it keeps you worry free and you may not get depression.”

CR2F is the best educated woman with 4 years of school. She is 62 years old and has lost all her children except one grown up son, whose daughter comes to help with cleaning and washing. She states that old people are treated as useless and clearly expresses her needs: “I hate being alone because it worries and I would like to have someone here to take care of me” and “When you don't have money, you tend to think a lot and also of the disease that killed my children” (CR2F). Here, we clearly see the need for the company in addition to the need for both financial and practical support.

The last of the care-receiving women, CR4F, has never attended school. She is 52 years old and has seven children with two men. She grew up in a household, where she was her mother's only child while she was among 30 of her father's children. She feels misunderstood by her community as she states that she got HIV from her brother, who purposely mixed some of his infected blood into her food. She did not develop HIV due to sexual intercourse, but it seems people find it hard to believe. When she found out that she was infected, she tried to take her own life. Her husband, who is HIV negative, left her after her status was known. But as they have a son together, he was obliged to buy her a plot and build a house. It seems that she does not get the necessary support from her three sons and four daughters, which causes her worries: “If I had something from which I could earn a living, I guess I would not have much worry; but in my situation, I only have this one child whose father left and he does not even provide fees, because even the child that used to help me, now has financial issues and so it keeps me worried.” Left without financial support for herself and the school fees for the son, she is worried. She also mentions poverty as the main cause for people getting depressed and secondly “… not having children to take care of you and maybe having children who neglect you and also a spouse who mistreats you and you have no peace of mind.”

The dominant issue for the female care-receivers was children and their ability and willingness to support them, whereas the most important issue for the male care-receiver is to become a respected and integrated member of the community. What was experienced meaningful in their life thus seemed to differ and be gender specific based on their different positions and access to support, which makes up their functional ability.

In the global meaning system of all care-receivers, both the Christian God, witches and witchdoctors and ancestors, were important. Misfortune and madness might befall a person if she/he has not appeased the ancestral spirits, whereas evil-minded witches equally can be blamed for these calamities including death. In the case of witches, one must turn to witchdoctors who might help according to CR1F. However, if one should be ill and admitted to a psychiatric hospital, it is in God's hands. Their global meaning system can without problems contain two different spiritual systems, which help them to make meaning and understand occurring situations. Depending on which force is perceived as responsible for a specific situation remedy is sought from a supernatural power that is assumed capable of rectifying and restore “normality.” Despite this eclectic approach making up the global meaning system, CR2F seems to mainly embrace her Christian belief, which provides her with the meaning for the harsh conditions: “Well, I don't have those negative thoughts because I believe people exist to suffer.” Through this approach, the suffering of her and others becomes a meaningful and normal way of living beyond her control, which protects her from ruminating.

### Perspectives of Caregivers

The caregivers were aged between 32 and 52 years old and all were made their living by farming and sometimes selling products. The people they supported were all but one HIV positive needing medication. The oldest in the group (48 and 52) were taking care of their spouses, whereas the youngest (32 and 39) took care of their mother and the last (unknown age) supported a neighbor. The only man in the group was 52 years, who took care of his wife. Except for the youngest caregiver, an immigrant from Rwanda, all confessed to the same eclectic global meaning-system we found in the care-receivers, but with a slight overweight to the traditional African worldview. Only one respondent (CG3F) indicated that she prayed and that the fate of all human beings was part of a divine plan. They all stated that old people in general have a tendency to worry and overthink as they have experienced hardship and disappointments in life: “I think the older you get, the more likely you are to get depressed, because as you age, you get many things that frustrate you and make you worry” (CG5F). Poverty and “the virus” (CG1F) make you especially overthink and loose hope. However, it also might depend on the context: “It depends on the condition of living, if someone is in good condition, someone can even reach 70 years with a sound mind and no worries” (CG4M). The caregivers seem to agree that old people are given less attention than younger people, which seems to be in accordance with the care-receivers perception of being useless. Interestingly, in contrast to the KI's accentuation of respect as the foundation for a meaningful late life, none of the caregivers ever mentioned this aspect. While the care-receivers complained about not being taken care of by their relatives and neighbors, these respondents support as much as they feel possible for them and thus contribute to their functional ability of care-receivers. Especially, interesting is the man, who is elderly himself (52 years), taking care of his wife. Being not unusual for a man to have more than one wife or to remarry a younger woman, who can take care of him in late life (Najjumba-Mulindwa, 2003), this respondent is of particular interest as he takes responsibility for his only wife: “Well, I was young, and I got her, and we now have children.” Interestingly, this seems difficult to understand for the interviewer, who repeatedly refers to him taking care of “this old lady” and the man's mother, whereas the respondent consequently talks about his wife. This thoroughgoing peculiar misunderstanding may have its origin in the rarity of the situation. The respondent has an educational background of 7 years of school. He mentions both ancestors, witches, and witchdoctors, but never a Christian God in his way of understanding and finding meaning in illness and calamities. His global meaning system seems to be dominated by a traditional African worldview, whereas his measures to prevent depression are from an interpersonal realm and include distraction and social contact to avoid thinking:

I: Personally, what do you do to distract yourself from over thinking and worrying
C: Keeping yourself busy with work
I: What else?
C: Well I don't really have many thoughts

Distraction is also what this respondent thinks helps his wife and other people, who need help: “Being there for the person and supporting them through engaging in activities that may distract the person from too many thoughts.” It is unclear why the wife needs help as she has tested negative for HIV and has, according to the husband, not shown signs of depression. While this respondent is reporting that others go to witchdoctors, a shrine, or eventually a hospital, he is convinced that interpersonal relations are meaningful and healthy. The male respondent was different from the female respondents in being more positive and relying mainly on the power of interpersonal relations as meaningful. CG2F, for example, has not the same faith in interpersonal relationships as meaningful or trust in other people: “Usually when you have a problem you just handle it alone, because you never know who wishes you well….” She is the only participant explicitly claiming that she does not believe in witches and spirits, which might indicate that her mistrust must be based on past experiences. She is the youngest person in the entire sample and has never been in school and immigrated from Rwanda.

## Discussion

We interviewed 14 people about the situation of the elderly in 3 groups: KIs, care-receivers, and caregivers. All the KIs were simultaneously elderly being more than 50 years. Among the caregivers, only one person was above 50 years. The KIs gave extensive descriptions of the conditions of the elderly as well as the public opinions and general normative context. It was against this rich material the interviews of the care-receivers and caregivers that were poorer in words were analyzed. The KIs described emaciated conditions, where the elderly were especially affected by poverty and lacking access to health facilities and support. Respect was mentioned as central for the possibility of living a life, which could be perceived as meaningful. However, the notion of respect was retrieved only in the interview of the male care-receiver and in none of the female care-receivers or the caregivers. In contrast, the caregivers agreed that the self-perception of care-receivers as useless was supported by their neglect by the community. Meaning was for the female care-receivers perceived as having children, who were willing, and able to support and care for their parents and consequently improve their functional ability, which was concordant with the KIs statements. The dominating global meaning-system for all but one participant seemed to be a fusion of Christian and traditional African worldviews. In our study, remarkable differences were noticed between the conditions of men and women as women were reported to suffer harsher conditions affecting their functional ability and meaning-making, which is in line with the findings of, for example, Rishworth et al. (2020). The respondents in this study are trapped in chronic poverty that embraces intergenerational and durational dimensions, where especially widowed, disabled, women, and those living alone are prone to chronic poverty Najjumba-Mulindwa (2003). Besides, older women are much more likely than older men to suffer from disabilities in old age according to the Ministry of Gender Labour Social Development (2018). Widowed, sick, and poor female respondents were dependent on relatives' and others' mercy and willingness to support, which should be compatible with African culture, where the community is the main element of human existence (Verhoef and Michel, 1997). Lack of or unstable support, therefore, might have a huge impact on their meaning-making and psychological wellbeing. As remarriage is not allowed for older women, they will have a weaker safety net based entirely on informal networks:

In Uganda, a person's safety net is their extended family as well as their community. These relations make up the “kinship networks” which offer them financial, physical, and emotional support, and a person becomes vulnerable when these are weak. Care is reciprocal so one must often contribute and look after others in the hope of receiving care in old age (Ministry of Gender Labour Social Development, 2018, p. 6).

Women, consequently, suffer accumulated disadvantages and in a study by Rishworth et al. (2020), lower subjectively reported wellbeing was found among older women than men in Uganda. This is no surprise given that meaning and wellbeing are strongly related, but dependent on the context:

Individuals' general tendency to use autobiographical memory for meaning making appears to be positively (and not negatively) related to SWB [Subjective well-being], but the exact nature of such relations varies by the component of meaning assessed, as well as by individuals' life phase and their cultural context (Alea and Bluck, 2013, p. 59).

The respondents in this study seemingly are captured in a situation of trans-generational chronic poverty Najjumba-Mulindwa (2003) without hope for change. The meaning-making activity is concentrated in the social area and the informal networks, where male and female respondents apparently are met differently. In 1998, Keyes defined five dimensions making up social wellbeing, where the social area is of special importance: social integration, social contribution, coherence, actualization, and acceptance. While respect was mentioned as fundamental by the KIs for a meaningful late life, it is interesting that only the male care-receiver feels that he receives sufficient respect and is socially integrated, neither the female care-receivers or any of the caregivers mention this aspect. Most models on wellbeing include a component of meaning and purpose (i.e., Baumeister, 1991; Wong, 2010, 2011, 2014). Especially, interesting is Baumeister's definition of meaning in life, whose basis is that four fundamental needs are met: purpose, efficacy or control, value and justification, and finally self-worth (Baumeister, 1991).

The care-receivers in our study might seem quite typical for this particular impoverished context as the male care-receiver had an established network, got his basic needs covered, and perceived social recognition as especially meaningful for him. He perceived to get the “required respect” and had some sense of efficacy by being asked for advice from others, which probably nourished his self-worth and sense of purpose and value. The women, on the other hand, had no network and were dependent on a sporadic support. Their situation was marked by unpredictability and a lack of means to cover their basic needs. The women felt useless and the caregivers supported their perception of the public opinion of them. The women thus seemed not to be able to meet Baumeister's fundamental needs for meaning in life.

According to the Ministry of Gender, Labour and Social Development, “older persons are also prone to social exclusion, in which the most vulnerable are ostracized by their family and the community. This is often targeted toward widowed women and in extreme cases can lead to witchcraft accusations” (Ministry of Gender Labour Social Development, 2018, p. 20). As the Ministry of Gender, Labour and Social Development underlines the reciprocity of care in the informal safety net, where one cares for others hoping that oneself will be cared for in late life, the hope of care has not been fulfilled for these older women and they feel useless. One might speculate whether these feelings mirror a self-perception of not being worthy to receive necessary support or care and consequently could be interpreted as self-reproach. This would be in line with the KIs' statements of the public opinion, where people might be blamed for their own misery as they assumingly have not prepared for old age. The feeling of uselessness simultaneously indicates that being useful is meaningful in the meaning system of the respondents. It is not described which usefulness they have in mind whether it is to contribute to the community and family actively or by being able to care for themselves and not being a burden for the community. Often described in African ethics is the necessity of contributing to the community: “Every member is expected to consider him/herself an integral part of the whole and to play an appropriate role towards achieving the good of all” (Gbadegesin, 1991, p. 65). The feeling of uselessness consequently might arise when one is dependent on others for care and support. In an emaciated context, where HIV/AIDS and rapid urbanization have weakened or disrupted the traditional, informal safety networks, this feeling as well as the public opinion of them being useless might be increased. Given the experience and sense of being useful seem especially meaningful for the care-receivers, the feeling of uselessness and the public opinion supporting this are alarming:

Sense of usefulness, feeling of social disconnectedness, and psychological pain associated with chronic physical illness should be assessed by practitioners who take care of older adults because of their strong association with suicidal behavior (Conejero et al., 2018, p. 697).

In an emaciated context without access to and means for basic needs and health facilities, the lack of experiencing a meaningful late life with all its dimensions mentioned above might have fatal consequences. As Uganda has no public statistics, we do not know whether Uganda follows the global trend of higher rates of suicide in older people (Conejero et al., 2018). Suicide attempt being a criminal offense (Penal code act Cap. 120, p. 109, Section 210), one could suspect that eventual suicides or suicide attempts among old people, who are regarded as useless, not necessarily would be reported. Especially this would be the case, where the elderly are perceived as an unnecessary burden for relatives and community.

Healthy aging includes human rights such as integrity, dignity, freedom, and autonomy (World Health Organization, 2015), and the rights for all people with a particular form of functional limitation, as most older people have acquired, to live and be included in their community (UN, 2006). The concept of functional ability has got a central status in studies on healthy aging (Cesari et al., 2018). Functional ability comprises both the intrinsic capacity of the individual and relevant environmental characteristics as well as the interaction between those (ICF, 2001) as a foundation to obtain the human rights. WHO (2001) emphasizes the support of intrinsic capacity/individual resilience based on the ability of an individual for a positive adaptation in case of adversities and the upholding of wellbeing and meaning in life. Without the predictability of sufficient support and care the female care-receivers seemed not to be able to live a meaningful life in contrast to the male care-receiver. However, despite these conditions, one of the female care-receivers (CR2F) manages to be positive as she has handed her fate over to God. As mentioned earlier, Uganda is a very religious country (Mbiti, 2006; Uganda Bureau of Statistics National Population Housing Census, 2014) and one could expect this to have an impact on the meaning-making of these elderly people as dominant belief systems like religions seem to be helpful for many people being comprehensive meaning systems (Hood et al., 2005; Silberman, 2005; Newton and McIntosh, 2013) offering meaning and hope in a variety of situations (Hall and Hill, 2019). Park (2010) describes that the global meaning system comes into action in the confrontation with potentially stressful events, and according to Hall et al. (2018) religion might contribute to coping in the context of encountering complex life events. However, reductionist Western meaning models seem inadequate to capture the cultural complexity of religions entirely and thus might have insufficient explanatory potential (Knizek et al., 2021). Both the KIs and most of the other respondents refer to an eclectic approach with a fusion of Christianity and traditional African belief systems and all but one referred to both religion and a traditional spiritual belief system in a meaning-making sense as explanatory tools for the prevailing conditions. Therefore, it is surprising that only one of the care-receivers mentions religion as a source for a meaningful life, hope, and solace, which protects her from rumination. Among the caregivers, also one female respondent put her trust into God, both for help and protection, whereas all the KIs unanimously assured that everything is part of a divine plan. Given that religion has been described as permeating all aspects of life in Africa (Mbiti, 2006), where “the African lives in a religious universe: all actions and thoughts have a religious meaning and aspired or influenced by a religious point of view” (Gyekye, 1995, p. 3), it is astonishing that so few care-receivers and caregivers mention religion even in a small study like this. In the absence of the fulfilment of all the basic needs for meaning in life, the individuals will feel distressed and frustrated and look for new sources of meanings for their lives (Baumeister, 1991). The women had no chances or hope for improving their situation, but they had clear ideas about what would have been a meaningful life for them. They did not look for new sources of meanings for their lives not even in religion. For them, a meaningful life was related to the predictability and coverage of basic needs, which again was related to their children as other relatives, who might have been able to care for their personal needs, might not be allowed due to strict norms. Emphasis on descendants is in line with children being valuable in the most high fertility settings of Africa and especially among the poor as they are perceived as old age security under pervasive insecurity conditions (Cain, 1985; Ntozi, 1995; Najjumba-Mulindwa, 2003). The changed social conditions indicate that governmental measures are needed to secure dignified and healthy aging in the sense of human rights as the informal, traditional networks have outlived their role in modern Uganda. Stable basic incomes with a fair distribution between men and women in need would improve the living conditions and reduce the danger of social exclusion and provide the possibility of living a meaningful life in old age. Improved access to necessary and affordable healthcare also is a crucial factor. The introduction of the Senior Citizen Grant is a laudable start but must be speeded up rapidly and improved significantly so that it reaches all old men and women that are in need equally. The functional ability and resilience of old people can be supported by the provision of the basic needs, which might give them the opportunity to make and find meaning in late life in Uganda.

### Limitations

This is a small study in a specific area. More participants in the care-receiver group would strengthen the study. In addition, participants from different areas, both rural and urban, would be valuable. As all care-receivers were HIV-infected and needed special care, a more heterogeneous sample might give a more nuanced picture. Spending some time in the field in advance with the participants might have improved the quality of the interviews, which partly were sparse in words. This study can, however, be regarded as a pilot study.

## Conclusion

In extreme poverty conditions, where all thoughts are bound to the coverage of the most basic needs, the question arises whether the perception of living a meaningful life that increases the functional ability is possible. While the KIs emphasized respect to be crucial tended to blame old, dependent people for their misery as a result of unsuccessful preparation for old age, the female care-receivers and the caregivers never mentioned this aspect. The female care-receivers felt useless, which also was a public opinion supported by the caregivers. Poverty, death of younger people, and rapid urbanization have affected the traditional, informal family and community-based networks severely, and left many old people in untenable situations without the possibility of living a life that is perceived as meaningful. Respect seemed especially to be related to financial independence/stability. The development and fair distribution of the Senior Citizen Grant guaranteeing the coverage of basic needs in later life are crucial in addition to the measures to restore dignity through a reintegration in both community and congregations to strengthen meaning in later life in Uganda.

## Data Availability Statement

The datasets presented in this article are not readily available because of ethical reasons as confidentiality was guaranteed as a precondition for the participants' consent. Requests to access the datasets should be directed to James Mugisha, jmmugi77@hotmail.com.

## Ethics Statement

The studies involving human participants were reviewed and approved by Mengo Hospital Research Ethics Committee. Written informed consent for participation was not required for this study in accordance with the national legislation and the institutional requirements.

## Author Contributions

All authors listed have made a substantial, direct and intellectual contribution to the work, and approved it for publication.

## Conflict of Interest

The authors declare that the research was conducted in the absence of any commercial or financial relationships that could be construed as a potential conflict of interest.

## Publisher's Note

All claims expressed in this article are solely those of the authors and do not necessarily represent those of their affiliated organizations, or those of the publisher, the editors and the reviewers. Any product that may be evaluated in this article, or claim that may be made by its manufacturer, is not guaranteed or endorsed by the publisher.
